# Pharmacological effects, classification, genetic and molecular studies of different chemotypes essential oil of *Perilla frutescens* (L.) Britt.: A review

**DOI:** 10.1016/j.jpha.2025.101454

**Published:** 2025-09-17

**Authors:** Wei Wei, Zhaoyuan Li, Bin Wang, Yang Liu, Yuxuan Sun, Dong Wen, Mei Rong, Pengcheng Huang, Yuwei Guo, Qiuling Wang, Zhihui Gao, Jianhe Wei

**Affiliations:** a Key Laboratory of Bioactive Substances and Resources Utilization of Chinese Herbal Medicine, Ministry of Education & National Engineering Laboratory for Breeding of Endangered Medicinal Materials, Institute of Medicinal Plant Development, Chinese Academy of Medical Sciences & Peking Union Medical College, Beijing, 100193, China; b Hainan Provincial Key Laboratory of Resources Conservation and Development of Southern Medicine & Key Laboratory of State Administration of Traditional Chinese Medicine for Agarwood Sustainable Utilization, Hainan Branch of the Institute of Medicinal Plant Development, Chinese Academy of Medical Sciences and Peking Union Medical College, Haikou, 570311, China

**Keywords:** *Perilla frutescens* (L.) Britt., Chemotype, Pharmacological effects, Classification, Genetics, Molecular mechanism

## Abstract

*Perilla frutescens* (L.) Britton is a highly valuable medicinal plant known for a wide variety of chemotypes based on the components of its essential oils. The appropriate and secure medicinal use of *P. frutescens* has been severely limited due to the lack of chemotype classification standards and pharmacological studies. In this paper, a classification standard for essential oils in *P. frutescens* was proposed. The pharmacological activities of different chemotypes or their main components were summarized. Genetic and molecular studies related to the formation of different chemotypes were also reviewed. The molecular mechanism underlying the formation of *P. frutescens* chemotypes is also discussed to pave the way for the innovation of *P. frutescens* germplasms for medicinal use.

## Highlights

• *Perilla frutescens* (L.) Britt. have abundance chemotypes variation of essential oils.
• Different chemotypes shows different pharmacological action.
• Cultivation based on chemotype can ensure the safety of *P. frutescens* medicinal use.
• Genetic and Molecular Studies pave the way for *P. frutescens* germplasms innovation.

## Graphical abstract

**Figure:**
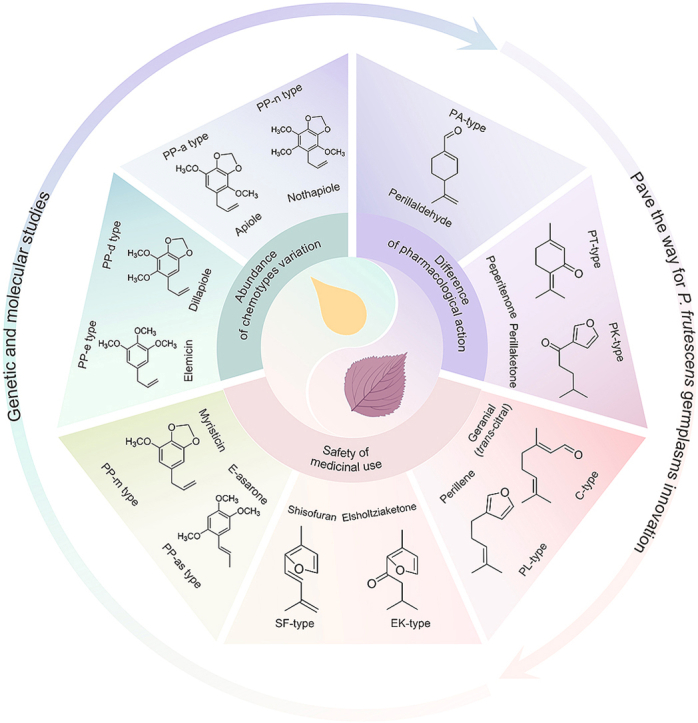

## 1 Introduction

*Perilla frutescens* (L.) Britton is an annual herb belonging to the Labiatae family, and it has been extensively used as an herbal medicine (medicine name Perilla) and vegetable in East Asia, particularly in China with a long history. The earliest historical record of *P. frutescens* dates back to the Western Han dynasty (202 BCE-220 CE), with a cultivation history of over 2000 years in China. *P. frutescens* is also popular in Southeast Asia, including in Vietnam [1], Laos [2], and Thailand [3]. It was introduced to various parts of Europe, such as Lithuania [4].

As herbal medicine, *P. frutescens* has broad effects on a series of diseases. In China, it was registered in the “Compendium of Materia Medica” (Li Shizhen, the Ming dynasty), and used for expelling wind dispersing cold, activating Qi circulation to harmonize the middle energizer, and treating fish and crab poisoning. Nowadays, its application includes the treatment of various illnesses, which have symptoms such as fever, chills, headache, stuffy nose, cough, chest congestion, vomiting, abdominal bloating and pain, constipation, and food poisoning from fish and crabs [5]. The plant is also used for similar diseases in other countries. For example, it is used to treat digestive disorders in India [6] and in Thailand, and its leaves are used in the form of a hot infusion (tea) to cure cold [7]. In addition, the juice of its fresh leaves can also be used to treat injuries in Nepal [8]. During the coronavirus disease 2019 (COVID-19) pandemic, it was used to alleviate the symptoms caused by COVID-19 in Vietnam [1].

**Fig. 1:**
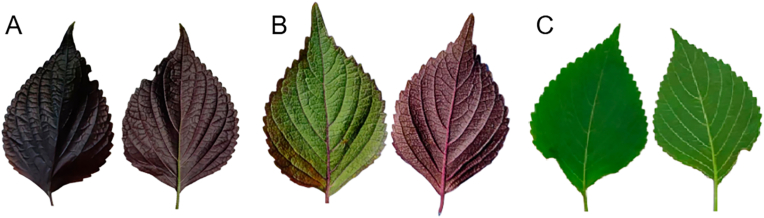
Different leaves color type of *Perilla frutescens*. (A) Double-sided purple. (B) Single-sided purple. (C) Double-sided green. By Figdraw.com.

*P. frutescens* varies a lot within a population. For example, *P. frutescens* has different leaf colors, and the Chinese Pharmacopoeia 2020 edition (https://ydz.chp.org.cn/) requires the *P. frutescens* leaves to be double-sided purple (Fig. 1A) or single-sided purple (Fig. 1B) while excluding double-sided green (Fig. 1C) leaves. With advancements in chemical analysis techniques, the presence of many different chemotypes in the essential oils of *P. frutescens* has been reported by researchers since the 1970s. These chemotypes each contain only one or two main constituents in their essential oils.

The studies on the mechanisms of the formation of this germplasm diversity were mainly focused on the leaf colors.

**Fig. 2:**
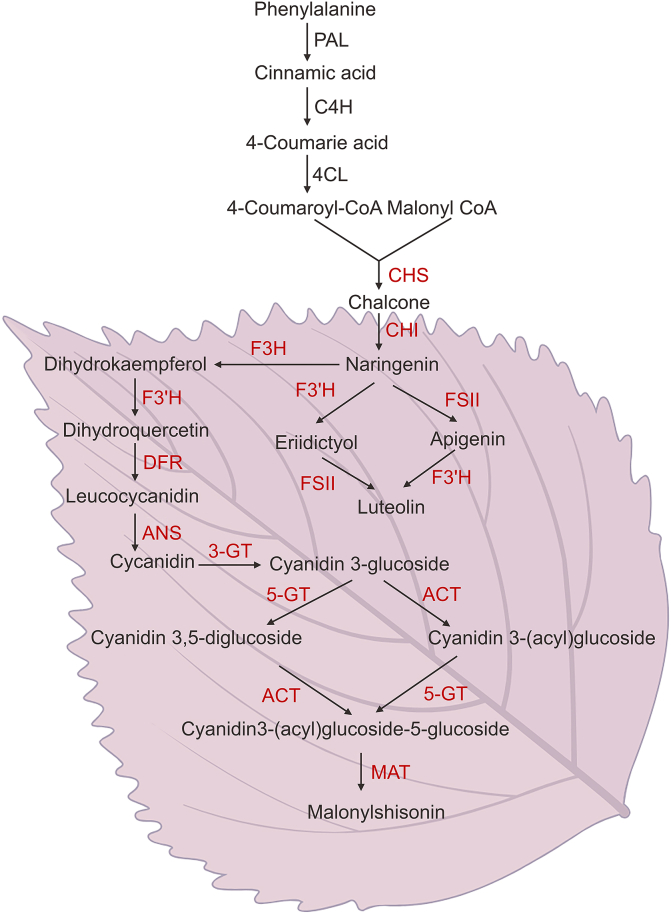
Biosynthetic pathway of flavonoids in the leaves of *Perilla frutescens*. The purple background indicates the anthocyanin biosynthetic pathway; red denotes the genes that have been identified in *P. frutescens*. PAL: phenylalanine ammonia-lyase; C4H: cinnamate 4-hydroxylase; 4CL: 4-coumarate-CoA ligase; CHS: chalcone synthase; CHI: chalcone isomerase; FSII: flavone synthase II; F3H: flavanone 3-hydroxylase; F3′H: flavonoid 3′-hydroxylase; DFR: dihydroflavonol-4 reductase; ANS: anthocyanidin synthase; 3-GT: flavonoid 3-O-glucosyltransferase; AAT: anthocyanin acyltransferase; 5-GT: anthocyanin 5-O-glucosyltransferase; MAT: malonyl-CoA: ACP transcasylase. By Figdraw.com.

Many studies have reported that the color of both the leaves and stems of *P. frutescens* is determined by the anthocyanin content [9,10]. Most genes responsible for anthocyanin biosynthesis have been isolated and characterized from *P. frutescens* [[11], [12], [13], [14], [15], [16], [17]] (Fig. 2).

However, studies related to chemotypes have been overlooked for a long time. For example, standard classification of chemotypes based on broad investigations is lacked. The relationships of the chemotypes to their safety and efficacy are not known. Additionally, the mechanisms underlying chemotype formation are not clear. Thus, the safe and secure medicinal use of *P. frutescens* and the efficient assessment of its germplasm resources are severely restricted.

Although lack of studies, the chemotypes have influenced the use of *P. frutescens*. For example, the double-sided green leaves of *P. frutescens* plant which is perilla ketone (PK)-type were used in Korea and Japan as a fresh vegetable and often accompanied with sashimi or grilled meat as a garnish, while the purple perillaldehyde (PA)-type leaves used as medicine. In China, PA is the chemical reference substance derived from *P. frutescens* leaves, as stipulated in the Chinese Pharmacopoeia 2020 version. Moreover, according to the quality standards of essential oils of *P. frutescens* leaves in Huoxiang Zhengqi oral solution, perillene (PL) content should not be less than 20% and PA content should not be less than 25 %. Thus, the identification of chemotypes is of great importance in the medicinal use of this plant.

Several chemotype varieties of *P. frutescens* with similar phenotypes are widely cultivated in mixture in China. The accurate chemotypes of them need to be identified, and the pharmacological activities and biosynthetic and regulatory mechanisms of those vital compounds need to be investigated, as these are the keys to cultivate high-quality and safe varieties of *P. frutescens* for medicinal use.

Here, we presented a comprehensive classification system for Perilla chemotypes and reviewed pharmacological and genetic studies on these different varieties. Moreover, the key genes identified in the biosynthesis pathways of different components were also summarized. This review set the stage for pharmacological, molecular and genetic studies.

## 2 Chemotype classification of essential oils in *P. frutescens*

Different methods, including steam distillation, hydro distillation, solvent extraction, Soxhlet extraction, and cold pressing, have been used to extract essential oils, which usually exist at low concentrations in plants [18]. In pharmacopoeia of China (2020 edition), essential oils are extracted from *P. frutescens* leaves by steam distillation, and their components were mostly identified by gas chromatography-mass spectrometry (GC-MS) analysis.

About 400 bioactive compounds have been isolated and identified in *P. frutescens*, including 193 volatiles identified via GC-MS analysis [19]. These compounds are predominantly monoterpenes, such as D-limonene, PL, PK and PA; however, some aromatic compounds, such as apiole, alpha-asarone, elemicin and myristicin were reported. It is noteworthy that many of these compounds exist in different individuals of the same Perilla species but not in the same individual. For example, it is very difficult to find both PL and PA in the same plant.

To facilitate the study of chemical constituents, researchers introduced the concept of the chemotype. Perilla plants were classified into different chemotypes by researchers in Japan based on the differences in main components of their essential oils. Firstly, the *P. frutescens* plants were divided into two groups: monoterpene (MT) and phenylpropylene (PP). MT-type *P. frutescens* could be further divided into seven subtypes: PA-type, PK-type, citral (C)-type, PL-type, piperitenone (PT)-type, shisofuran (SF)-type, and elsholtzia ketone (EK)-type [20]. The PP type, however, was categorized into five subtypes. The PP-m type was mainly composed of myristicin, whereas dillapiole and myristicin were the primary constituents of the PP-dm type, and the main components of the PP-em type were elemicin and myristicin. Additionally, the PP-dem type generally contained dillapiole, elemicin, and myristicin, and the PP-dmn type mainly consisted of dillapiole, myristicin, and nothoapiole [21]; thus, a total of eight chemotypes (seven MT types, including the PA-type, PK-type, C-type, PL-type, PT-type, SF-type, and EK-type, and all PPs were considered one type designated as the PP type) were identified. Next, DLP types consisting of D-limonene and piperitone were reported [22]. The main component of PT-type is piperitenone. The main component of MS-type is the myristicin, the main component of AL-type is the apiole, the main component of EM-type is the elemicin, and the main component of dehydroelsholtzia ketone (DEK)-type is the dehydroelsholtzia ketone [22]. The widespread distribution of PAPK-type and PKPA-type chemotypes in China, which mainly include PA and PK, was reported in a study [23] (Table 1). Although not confirmed in genetic studies of chemotypes, this kind of mixed chemotypes will provide convenience for the quality control and production of related medicines, because medicines such as previously mentioned Huoxiangzhengqi oral liquid requires the content of both PL and PA.

**Table 1:** The chemotype classification of monoterpenes (MTs) in *Perilla frutescens*.

No.	Pure/Mix	Chemotype name	Characteristic compound	Refs.
1	Pure	EK	The main content is elsholtziaketone.	[20]
2	Pure	PA	The main content is perillaldehyde.	[21]
3	Pure	PK	The main content is perilla ketone and/or isoegomaketone.	[21]
4	Pure	PL	The main content is perillene.	[21]
5	Pure	C	The main content is citral.	[21]
6	Mixed	PAPK	Both perillaldehyde (more) and perilla ketone.	[23]
7	Mixed	PKPA	Both perilla ketone (more) and perillaldehyde.	[23]
8	Pure	SF	The main content is shisofuran.	[26]
9	Pure	PT	The main content is piperitenon.	[27]

PA: perillaldehyde; PK: perilla ketone; PL: perillene; C: citral; SF: shisofuran; EK: elsholtziaketone; PT: piperitenone.

However, chemotypes were not clearly defined until 2014. Chemotypes was defined as organisms belonging to the same species, subspecies, or varieties, with differences in the quantity and quality of the chemical composition of their fingerprint, which can be inherited [24]. No clear requirement for the proportion of its main components was mentioned in previous studies [21]. Additionally, some naming errors were made in the classification of the chemotype *P. frutescens* by different researchers. For example, DEK was a main compound of DEK-type, is the same as naginata ketone, which was an compound in EK-type, as proposed in a study [21]. DEK should also be designated as the EK-type since DEK acts as a precursor of EK. Similarly, D-limonene is the precursor of piperitone, thus, DLP should be referred to the PT-type. In addition, The AL-type of apiole in the PP group should be referred to the PP-a type, the EM-type should be referred to the PP-e type, and the MS-type should be referred to as the PP-m type.

**Fig. 3:**
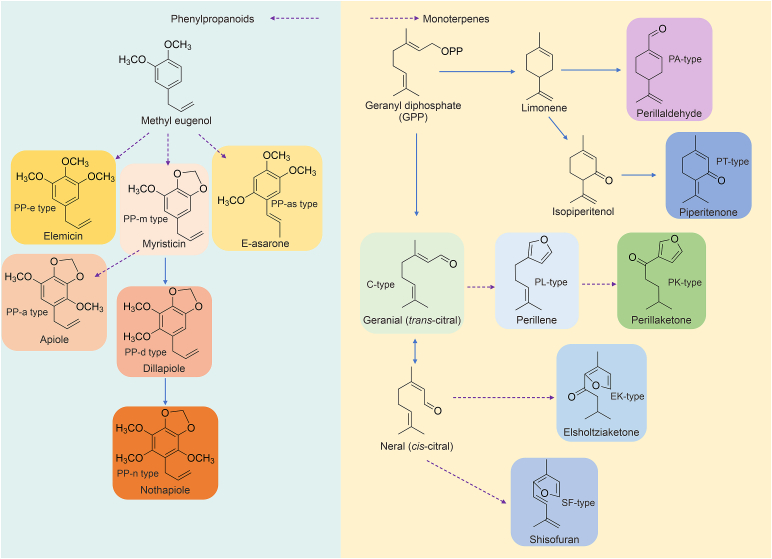
The relationships between different chemotypes in *Perilla frutescens*. PA: perillaldehyde; PK: perilla ketone; PL: perillene; PT: piperitenone; C: citral; EK: elsholtziaketone; SF: shisifuran; PP-as: (E)-asarone; PP-n: nothoapiole; PP-m: myristicin; PP-d: dillapiole; PP-e: elemicin; PP-a: apiole. By Figdraw.com.

Here, we followed the criteria for chemotype classification, which is based on the proportion of different compound contents. Pure (well-defined) chemotypes are characterized based on a different dominating component that accounts for more than 50% of the complete chemical composition. On the other hand, a mixed chemotype is defined by 2–3 main compounds, each representing less than 50% of the total composition [25]. Based on these criteria, all reported chemotypes have been summarized here (Table 1, Table 2). Those naming errors and naming conflicts have been corrected. The DEK-type was renamed as the EK-type, the DLP-type was renamed as the PT-type, the MS-type was renamed as the PP-m type, the EM-type was renamed as the PP-e type, and the AL-type was renamed as the PP-a type. The relationships among the different chemotypes of *P. frutescens* are shown in Fig. 3.

**Table 2:** The chemotype classification of phenylpropylenes (PPs) in *Perilla frutescens*.

No.	Pure/Mix	Chemotype Name	Characteristic compound	Refs.
1	Pure	PP-e	The main content is elemicin.	[20]
2	Pure	PP-m	The main content is myristicin.	[20]
3	Pure	PP-d	The main content is dillapiole.	[20]
4	Pure	PP-a	The main content is apiole.	[22]
5	Pure	PP-n	The main content is nothoapiole.	[28]
6	Mixed	PP-dm	Dillapiole and myristicin.	[28]
7	Mixed	PP-em	Elemicin and myristicin.	[28]
8	Mixed	PP-dem	Dillapiole, elemicin and myristicin.	[28]
9	Pure	PP-as	The main content is (E)-asarone.	[29]
10	Mixed	PP-mdn	Myristicin, dillapiole and nothoapiole.	[30]
11	Mixed	PP-dmn	Dillapiole, myristicin and nothoapiole.	[31]

PP-m: myristicin; PP-d: dillapiole; PP-a: apiole; PP-e: elemicin; PP-n: nothoapiole; PP-as: (E)-asarone; PP-dm: dillapiole and myristicin; PP-em: lemicin and myristicin; PP-dem: dillapiole, elemicin and myristicin; PP-mdn: myristicin, dillapiole and nothoapiole; PP-dmn: dillapiole , myristicin and nothoapiole.

First, the chemotypes of *P. frutescens* could be divided into MT and PP types according to the chemical framework of the main compounds of the essential oils [26]. For MTs (Table 1), based on their principal constituents, pure chemotypes could be further categorized, including types C [21], EK [20], PA [21], PK[21], PL [21], PT [27], and SF [26]. The mixed chemotypes were PAPK and PKPA [23]. For PPs (Table 2), pure chemotypes were classified into different groups, including PP-e [20], PP-m [20], PP-d [20], PP-a [22], PP-n [28], and PP-as [29], whereas PP-dm, PP-em, PP-dem [28], PP-mdn [30] and PP-dmn [31] were the subcategories of mixed PP chemotypes.

## 3 Pharmacological effects of main components in essential oils of different chemotypes

**Fig. 4:**
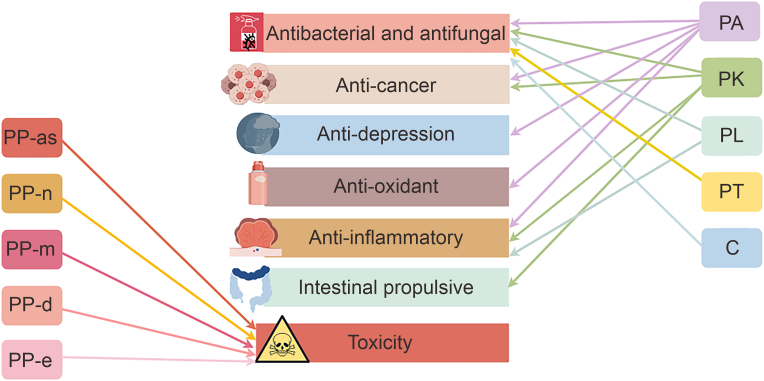
The correlations between pharmacological effects and the main compounds in different chemotypes of *Perilla frutescens*. PA: perillaldehyde; PK: perilla ketone; PL: perillene; PT: piperitenone; C: citral; PP-as: (E)-asarone; PP-n: nothoapiole; PP-m: myristicin; PP-d: dillapiole; PP-e: elemicin. By Figdraw.com.

Pharmacological studies have revealed various biological activities, such as antioxidant, anti-inflammatory, antibacterial, antifungal, antidepressant and anticancer activities of the main components of different chemotypes of *P. frutescens*. The different pharmacological effects of the main components from essential oils of different chemotypes can differ (Fig. 4). The classified use of different chemotypes of Perilla can make its medicinal use more precise and effective. Therefore, the pharmacological effects of the components found in different chemotypes of *P. frutescens* need to be determined (Table 3, Table 4).

**Table 3:** Pharmacological effects of the main components of different chemotypes of *Perilla frutescens*.

Chemotype Name	Compounds	Activities	Refs.
C	Citral	Antimicrobial and antifungal activity	[35]
PA	Perillaldehyde	Antimicrobial and antifungal activity	[[32], [33], [34], [35]]
		Antioxidant activity	[39,40]
		Anti-inflammatory activity	[41]
		Antidepressant-like effects	[[45], [46], [47], [48], [49]]
		Anti-cancer activity	[50,51]
PK	Perilla ketone	Antimicrobial and antifungal activity	[36,37]
		Intestinal propulsion	[55]
	Isoegomaketone	Anti-inflammatory activity	[[42], [43], [44]]
		Anti-cancer activity	[52,53]
PL	Perillen	Antimicrobial and antifungal activity	[38]
		Anti-inflammatory activity	[38]

C: citral; PA: perillaldehyde; PK: perilla ketone; PL: perillene.

**Table 4:** Toxicology of some main components of essential oils from specific chemotypes.

Chemotype Name	Compounds	Toxicology	Refs.
PK	Perilla ketone	Acute pulmonary emphysema in livestock	[56]
PP-as	(E)-asarone	Carcinogenic	[57,58]
PP-m	Myristicin	Genotoxic and carcinogenic	[59]
PP-d	Dillapiole	Genotoxic and carcinogenic	[59]

PK: perilla ketone; PP-as: (E)-asarone; PP-m: myristicin; PP-d: dillapiole.

### 3.1 Antibacterial and antifungal activities

The component PA of essential oils in PA-type *P. frutescens* could inhibit bacterial reproduction and prevent food spoilage [32,33]. The antibacterial activity of *P. frutescens* essential oil was evaluated using *Enterococcus faecalis* R612-Z1 as the target strain. The minimum inhibitory concentration of PA-type *P. frutescens* essential oil against *E. faecalis* was 0.5 μL/mL [32]. The ability of PA to decrease the growth of *Candida albicans,* which causes oral inflammation, has also been demonstrated [34]. Low concentrations of PA inhibited biofilm formation, prevented the transition to the mycelial phase, and decreased the expression of secreted aspartic proteinases (SAPs) genes of *C. albicans in vitro*. Moreover, in a study on the inhibition rate against airborne microbes, PA was found to have the highest average inhibition rate of 53%, followed by trans-cinnamaldehyde at 45%, carvacrol at 34%, citronella at 30%, citral at 17%, and eugenol at 13% (Table 3) [35]. So the PA-type *P. frutescens* essential oil and PA both have antibacterial and antifungal activities.

The main component PK of essential oils in PK-type *P. frutescens* has antifungal effects. It could affect biofilm formation in various fungi, includ*ing Colletotrichum musae, Fusarium dimerum and Fusarium oxysporum*, reduce conidia adhesion and germination, and hinder the development of structural biofilms [36]. PK could affect the surface sensing mechanism of fungi [37] by activating the highly conserved transient receptor potential (TRP) channel to exert antifungal activity. Moreover, PL of PL-type *P. frutescens* exhibited antibacterial activity against *Staphylococcus aureus* and *Escherichia coli* (*E. coli*) [38]. According to these studies, almost all investigated components in essential oils had antifungal and antibacterial activities, and this function of PA was stronger than that of other compounds, such as citral in the C-type *P. frutescens*.

### 3.2 Antioxidant activity

PA is a novel thioredoxin inducer. Pretreatment with PA could decrease H_2_O_2_-induced cytotoxicity [39]. In a study in which the human keratinocyte cell line HaCaT was used, PA inhibited benzo(a)pyrene (BaP)-induced aryl hydrocarbon receptor (AHR) activation and reactive oxygen species (ROS) production, inhibited BaP-mediated and AHR-mediated release of the C-C motif chemokine ligand 2 (CCL2) chemokine, and activated the nuclear factor-erythroid 2-related factor-2 (NRF2) and heme oxygenase-1 (HO1) antioxidant pathways (Table 3) [40].

### 3.3 Anti-inflammatory activity

In a model of mice, PA could attenuate serum interleukins (ILs) and tumor necrosis factor-α (TNF-α) levels, as determined by enzyme-linked immunosorbent assay (ELISA) of PA treatment. This finding indicated that the Nrf2 and HO-1 signaling pathways contributed to the anti-inflammatory effect of PA [41].

The isoegomaketone from the PK chemotype of *P. frutescens* had anti-inflammatory properties [42]. Radiation-induced mutants of *P. frutescens* with relatively high levels of isoegomaketone could efficiently relieve arthritis pain, with greater therapeutic effects than wild-type plants [43]. Isoegomaketone compounds were used to study the *in vitro* anti-inflammatory activity in mouse monocyte-macrophage RAW 264.7 cells and it had high activity against the inflammatory factors nitric oxide (NO), monocyte chemotactic protein-1 (MCP-1), and interleukin-6 (IL-6) [44].

Isoegomaketone regulated gene transcription via nuclear factor kappa-B (NF-κB) and activator protein (AP-1) by reducing their transcriptional activity [42]. PL also had anti-inflammatory activity [38]. To summarize, PA, PL, and isoegomaketone from PK-type *P. frutescens* have been found with anti-inflammatory effects (Table 3).

### 3.4 Antidepressant activity

A study reported the antidepressant-like activity of PA via olfactory nervous system functioned by inhalation [45]. The forced swimming test was performed to measure the duration of mobility of the model mice, which revealed that the inhalation of PA (0.0965 and 0.965 mg/mouse/day, nine days) significantly shortened the duration of immobility of the model mice, whose stress was induced by the combination of forced swimming and chronic mild stress. Additionally, the antidepressant-like activity of PA in treating lipopolysaccharide (LPS)-induced depressive-like behavior in mice has been reported [46]. PA administration also alleviated depression-like behavior in a rat model induced by chronic unpredictable mild stress (CUMS). PA could inhibit the reduction and depletion of 5-hydroxytryptamine (HT) and norepinephrine (NE) in the prefrontal cortex, which might be related to its antidepressant function [[47], [48], [49]]. However, other chemotypes of *P. frutescens* were not reported to have antidepressant activity (Table 3)*,* except PA-type.

### 3.5 Anticancer activity

Both PA and PK have anticancer activities (Table 3). PA could suppress the growth of gastric cancer cells by inducing autophagy [50] and inhibit the proliferation, invasion, and migration of prostate PC-3 cancer cells. PA could also suppress osteoclast differentiation and bone-specific metastasis in RAW264.7 cells [51]. The isoegomaketone of the PK-type could induce apoptosis in DLD1 human colorectal cancer cells via a mitochondrial apoptosis-inducing factor (AIF)-dependent pathway by inducing the translocation of AIF from the inner and outer intermembrane spaces of the mitochondria into the nucleus [52]. Isoegomaketone could also significantly inhibit the activity of human hepatoma cells (HCCs), which exert anticancer effects by blocking the PI3K/Akt signaling pathway [53].

### 3.6 Other beneficial activities and potential toxicity

Inconsistent results concerning the toxicity of Perilla have been obtained from other studies, and it has not been sufficiently investigated, with only a few reports indicating its potential toxicity (Table 4).

PA has been granted “generally recognized as safe” (GRAS) status by the United States Food and Drug Administration (FDA), and the Expert Panel of the U.S. Flavor and Extract Manufacturers Association (FEMA) judged PA is also safe for use as a condiment in everyday foods as it does not have genotoxic properties or unknown harmful effects on humans [54].

The perilla ketone of the PK-type *P. frutescens* exhibited intestinal propulsive activity and could stimulate the motility of intestinal circular muscles [55]. Besides showing pulmonary toxicity, it served as a potent pulmonary edemagenic agent for laboratory animals and livestock [56].

The toxicity of asarone, which belongs to the PP-as type, has been reported, among which (Z)-asarone isomer and (E)-asarone were toxic [57] and carcinogenic [58], respectively. Moreover, the genotoxicity and carcinogenicity of myristicin from the PP-m type and dillapiole from the PP-d type have been reported [59]. These toxic substances have severe toxic effects, therefore undivided attention should be paid to avoid the use of chemotypes PP-as, PP-m, and PP-d of *P. frutescens*.

Overall, the pharmacological functions of components of different chemotypes differ. Pharmacological studies have focused on the PA-type, whereas few studies have investigated the PK-type and PL-type. Although, activities mentioned above have been proved by experiment *in vitro*. However, the effects of different components still need to be further verified by clinical trials and pharmacokinetics experiments [60].

## 4 Genetic studies of different chemotypes of *P. frutescens*

Why does *P.frutescens* have a wide variety of chemotypes, and what are the molecular and genetic mechanisms underlying their formation? Can mixed chemotypes of *P.frutescens* be derived from crossing? The molecular and genetic mechanisms of the formation of chemotypes are of great significance for cultivating superior varieties, promoting safety, and developing the potential use of *P. frutescens* as a medicine. Many studies have investigated the laws of inheritance in *P.frutescens*.

In the 1990s, Japanese researchers investigated the law of inheritance of different chemotypes in *P. frutescens* with F1 and F2 hybrid offspring resulting from crosses between different chemotypes of *P. frutescens*. No mixed chemotypes of *P. frutescens* were detected in the F1 and F2 generation plants. The genetic segregation ratios in the F1 and F2 generations of different *P. frutescens* strains with different essential oil chemotypes were presented in Table 5 [[61], [62], [63], [64]].

**Table 5:** Different chemotypes of essential oils in parental lines of *Perilla frutescens* used for crossing.

Chemotype Name	F1	Genetic segregation of F2				Refs.
Cross (P_1_ x P_2_)	Chemotype	Chemotype	Observed	Expected ratio	P value of χ^2^ test	
PP x PA	PA	PA:PP	41:11	3:1	0.8	[61]
PK x PA	PA	PA:PK	46:9	3:1	0.2	[61]
PK x PA	PA	PA:PK	40:13	3:1	0.9	[61]
PK x EK	EK	EK:PK	36:6	3:1	0.1	[61]
PA x EK	PA	PA:EK	38:13	3:1	0.9	[61]
PK x PL	PK	PK:PL	65:22	3:1	0.95	[61]
PL x PK	PK	PK:PL	65:25	3:1	0.4	[61]
C x PL	PL	PL:C	44:15	3:1	0.9	[62]
PL x C	PL	PL:C	68:19	3:1	0.3	[62]
C x PA	PA	PA:C	25:13	3:1	0.5	[62]
C x EK	EK	EK:C	65:22	3:1	0.95	[62]
PK x PL	PK	PK:PL	65:22	3:1	0.8	[63]
PL x EK	EK	EK:PL	62:18	3:1	0.6	[63]
PT x PA	PA	PA:PT	64:24	3:1	0.5	[64]
PT x EK	PT	PT:EK	64:20	3:1	0.8	[64]
PT x PK	PT	PT:PK	61:19	3:1	0.7	[64]
PT x C	PT	PT:PL:C	68: 18:9	12:3:1	0.3	[64]

P_1_ and P_2_ represent the female and male parents, respectively. PP: phenylproplene; PA: perillaldehyde; PK: perilla ketone; EK: elsholtziaketone; PL: perillene; C: citral; PT: piperitenone.

The hybridization results revealed dominant-recessive relationships among the chemotypes. As shown in Table 5, PA-type is the dominant phenotype in the F1, when hybrid with PT, PP, PK, EK, C, either as paternal or maternal parents. Despite being relatively recessive in relation to the PA-type, the PK-type was dominant to the PL-type. Even without conducting the cross between PA-type and PL-type, it could be inferred that PL-type might also be a recessive phenotype if it is crossed with PA-type. Similarly, C-type was found to be recessive to PL-type, and thus, probably also to PK-type. These results indicated that the recessiveness of C-type to PA-type was rational. Thus, the laws of genetics of different chemotypes of *P. frutescens* could be proposed*.* PA-type was the most dominant phenotype, followed by PT-type, EK-type, PK-type, PL-type, and C-type.

The inheritance patterns of essential oil chemotypes in other plant species have been partially explored. A study of the genetic regulation of the oil content in *Thymus vulgaris* proposed a case of polymorphism in a biosynthetic chain [65]. For example, a strict epistasis chain was shown in the following order: G > A > U > L > C > T. This is similar to the explicit recessive inheritance of different chemotypes in *P. frutescens*.

The genetic laws of hybridization of different chemotypes were described in the 1990s. These inheritance results combined with compounds detected in chemotypes preliminarily form a network [62], laying a foundation for studies on genetic mechanisms underlying different chemotypes.

As to genetic mechanisms, owing to technical limitations, no study has investigated the recessive inheritance mechanisms of these chemotypes. The first genome of *P. frutescens* was reported in 2021, and *P. frutescens* was found to be an allotetraploid [66]. A genome assembly of *P. frutescens* of the PA-type was reported in 2023 [67]. Although none of these studies focused on the genetic mechanisms underlying the formation of different chemotypes, these studies provided a basis for future studies. Through metabolome and transcriptome analysis in combination with whole-genome resequencing, metabolome genome-wide association Study (mGWAS), and metabolome quantitative trait locus (mQTL) analysis, the mystery of chemotype inheritance in *P. frutescens* and molecular breeding of the medicinal *P. frutescens* species will be determined soon.

## 5 Biosynthetic pathways of different chemotypes of *P. frutescens*

**Fig. 5:**
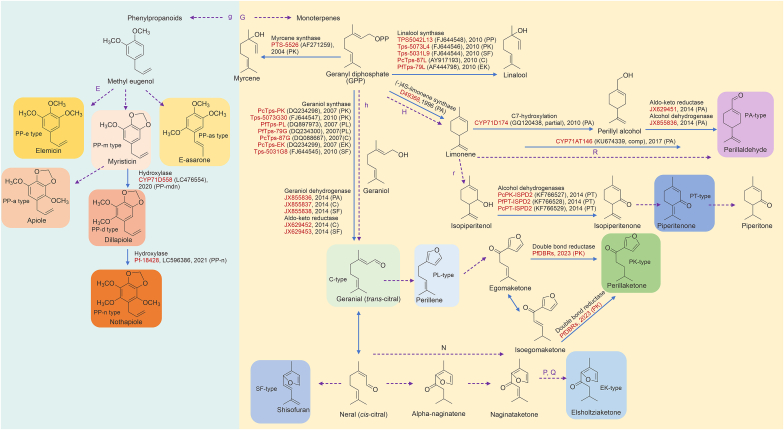
Hypothetical biosynthetic pathways of monoterpenoids (MTs) and phenylpropanoids (PPs) in *Perilla frutescens*. Bold letters and purple dotted arrows indicate the steps of putative genes involved in biosynthesis of these chemotypes that not been characterized functionally, whereas blue solid arrows denote the biosynthesis steps of genes that have been characterized functionally. PA: perillaldehyde; PK: perilla ketone; PL: perillene; PT: piperitenone; C: citral; EK: elsholtziaketone; SF: shisofuran; PP-as: (E)-asarone; PP-n: nothoapiole; PP-m: myristicin; PP-d: dillapiole; PP-e: elemicin; PP-a: apiole. By Figdraw.com.

Over the past 20 years, all genes encoding enzymes involved in the biosynthetic pathways for PA and C have been functionally characterized. The biosynthetic pathways of other chemotypes have not been fully characterized. Several studies have preliminarily outlined the biosynthetic pathways of essential oils in *P. frutescens* (Fig. 5, and Table 6, Table 7). Among them, the MT group primarily consists of monoterpenes, whereas the phenylpropene (PP) mostly comprises phenylpropane derivatives. However, many steps have not been clarified with certain enzymes (dotted arrows in Fig. 5).

**Table 6:** Key genes identified in the biosynthetic pathways for monoterpenoid (MT) chemotypes.

Chemotype name	Enzyme type	Enzyme name	GenBank ID	Year	Refs.
PA	Aldo-keto reductase	PfAKR	JX629451	2014	[26]
C	Aldo–keto reductase	PcAKR	JX629452	2014	[26]
SF	Aldo–keto reductase	PsAKR	JX629453	2014	[26]
PA	Geraniol dehydrogenase	PfGeDH	JX855836	2014	[26]
C	Geraniol dehydrogenase	PcGeDH	JX855837	2014	[26]
SF	Geraniol dehydrogenase	PsGeDH	JX855838	2014	[26]
PA	Limonene cyclase	–	D49368	1996	[70]
PA	Perilla (−)-limonene-7-hydroxylase	CYP71D174	GQ120438	2010	[71]
PA	Perillalcohol and perillaldehyde synthase	CYP71AT146	KU674339	2017	[72]
PT	Isopiperitenol dehydrogenases	PcPT-ISPD2	KF766528	2014	[73]
PL	Geraniol synthase	PfTps-PL	DQ234300	2007	[74]
EK	Geraniol synthase	PcTps-EK	DQ234299	2007	[74]
PK	Geraniol synthase	PcTps-PK	DQ234298	2007	[74]
C	Geraniol synthase	PcTps-C	DQ088667	2010	[75]
PK	Geraniol synthase	PhTps-5073G	FJ644547	2010	[75]
SF	Geraniol synthase	PsTps-5031G	FJ644545	2010	[75]
PA	Geraniol synthase	PfTps47-PA	None	2024	[76]

PA: perillaldehyde; C: citral; SF: shisofuran; PT: piperitenone; PL: perillene; EK: elsholtziaketone; PK: perilla ketone.

**Table 7:** Other related genes identified in the biosynthetic pathways for monoterpenoid (MT) and phenylpropanoid (PP) chemotypes.

Chemotype name	Enzyme type	Enzyme name	GenBank ID	Year	Refs.
C	Linalool synthase	PcLnS	AY917193	2007	[74]
PP	Linalool synthase	PhTps-5042L13	FJ644548	2010	[75]
PK	Linalool synthase	PhTps-5073L4	FJ644546	2010	[75]
SF	Linalool synthase	PsTps-5031L	FJ644544	2010	[75]
EK	Linalool synthase	PfTps-79L	AF444798	2010	[75]
PA	Linalool synthase	PfTps18-PA	None	2024	[76]
PA	Linalool synthase	PfTps49-PA	None	2024	[76]
PK	Linalool synthase	PfTps16-PK	None	2024	[76]
PL	Linalool synthase	PfTps46-PL	None	2024	[76]
PK	Myrcene synthase	PTS-5526	AF271259	2004	[80]

C: citral; PK: perilla ketone; SF: shisofuran; EK: elsholtziaketone; PA: perillaldehyde; PL: perillene.

### 5.1 Biosynthesis of MTs

Monoterpenes (MTs) were regarded as secondary metabolites produced via mevalonic acid in the mevalonate (MVA) pathway. Isopentyl diphosphate (IPP) and dimethyl allyl diphosphate (DMAPP) further constructed a monocyclic menthol skeleton by forming geranyl pyrophosphate (GPP) [68,69]. Terpene synthases (TPS) and CYP450 enzymes were involved mainly in the synthetic pathway of MTs.

Some researchers have hypothesized that the dominant gene H originally controls the initiation of the PA pathway [62] and the cyclic intermediate limonene. Enzymes in biosynthetic pathways of the PA-type have been functionally characterized. The (−)-4S-limonene synthase from *P. frutescens* (GenBank Accession No. D49368) was identified and functionally expressed in *E. coli* in 1996 (Table 6) [70], yielding an enzyme that was catalytically active in generating (4S)-limonene with GPP as substrate. The cyclization of GPP to form (4S)-limonene, followed by hydroxylation at the C7 position and oxidation to PA. The (−)-limonene-7-hydroxylase CYP71D174 (GenBank Accession No. GQ120438) isolated from PA-type *P. frutescens* in 2010 was identified by Mau (Table 6) [71].

Another cytochrome P450 CYP71AT146 (GenBank Accession No. KU674339), which catalyzed the oxidation of limonene to perillyl alcohol and subsequently to PA, was cloned by Fujiwara and Ito (Table 6) [72]. Other studies [20,64] proposed that the dominant gene R controlled the biosynthesis of the PA-type, whereas the PT-type was synthesized under the control of the recessive gene R.

For the enzymes in the biosynthetic pathway of PT-type, the isopiperitenol dehydrogenases 1 and 2 (ISPD1 and ISPD2) isolated from *P. frutescens* in 2014 participated in the biosynthesis of PT, whereas only PcPT-ISPD2 (GenBank Accession No. KF766529) played a role in the selective conversion of (−)-cis-isopiperitenol into (−)-isopiperitenone but not in the catalysis of the reverse reaction (Table 6) [73].

The critical step of terpenoid biosynthesis, which determines whether the terpenoid biosynthesis pathway is localized to skeleton of furylalkenes, such as EK, PK, PL, and SF, was catalyzed by the geraniol synthase enzyme, whereas limonene synthase catalyzes the formation of the cyclohexene skeleton.

Geraniol synthases (Table 6) PcTps-C (GenBank Accession No. DQ088667) and PfTps-PL (GenBank Accession No. DQ234300) were isolated from C-type *P. citriodora* and PL-type *P. frutescens*, respectively [74]. The heterologous expression of PcTps-C in *E. coli* and the subsequent enzymatic assay with purified His-tagged proteins revealed their roles in the transformation of GPP into geraniol, with divalent metal ions (Mn^2+^ and Mg^2+^) acting as cofactors. PcTps-EK (GenBank Accession No. DQ234299) and PcTps-PK (GenBank Accession No. DQ234298) isolated from EK-type and PK-type *P. citriodora*, respectively, were identical to PcTps-C at the nucleotide sequence level [62]. The geraniol synthase gene PhTps-5073G (GenBank Accession No. FJ644547) was isolated from PK-type *P. hirtella*, whereas the isolation of PsTps-5031G (GenBank Accession No. FJ644545) was performed from SF-type *P. setoyensis* [75]. In 2024, the geraniol synthase gene PfTPS47-PA was isolated from the PA-type of *P. frutescens* [76]. However, the functional identification of these genes has not been performed.

Geraniol dehydrogenase (GeDH) determines the formation of the C-type. GeDH could catalyze geraniol to geranial (trans-citral), the main constituent of essential oil from C-type Perilla. GeDH and aldo-keto reductase (AKR) were cloned from different chemotypes in 2014 [26] (Table 6). Aldo-keto reductase PfAKR (GenBank Accession No. JX629451), PcAKR (GenBank Accession No. JX629452), and PsAKR (GenBank Accession No. JX629453) were cloned from PA-type *P. frutescens*, C-type *P. citriodora*, and SF-type *P. setoyensis*, respectively. The geraniol dehydrogenase PfGeDH (GenBank accession no. JX855836) was isolated from PA-type *P. frutescens*, whereas the chemotype from which PcGeDH (GenBank Accession No. JX855837) was isolated was C-type *P. citriodora*. PsGeDH (GenBank Accession No. JX855838) was isolated from SF-type *P. setoyensis* in 2014 [26].

Both PsAKR and PcGeDH catalyzed the conversion of geraniol into citral and nerol and the conversion of perilla alcohol into PA, presenting greater substrate promiscuity than monoterpene synthases such as geraniol synthase. The genes encoding the AKR and GeDH enzymes both expressed in Perilla of different chemotypes.

C is the precursor of PL and PK. The pathway through which PL and PK are synthesized from C has not been fully elucidated. Since the menthofuran synthase of mint that catalyzes the formation of the furan ring is a cytochrome P450, the biosynthesis of PL from C may also be performed by a cytochrome P450 monooxygenase [77], which has not been identified and needs further assessment. The conversion of PL into egomaketone was speculated to be performed under the action of gene J, which was expected to be a cytochrome P450 monooxygenase [60]. The synthesis of perilla ketone further led to the formation of the PK-type. The biosynthesis of egomaketone, isoegomaketone, and PK has been investigated via cross-breeding. Using the isotope-tracer method, egomaketone was found to be the synthesis substrate for isoegomaketone, and PK and isoegomaketone could not be converted into PK [62,78]. However, a study [79] reported the transformation of isoegomaketone to PK by double bond reductases (PfDBRs) in *P. frutescens*, suggesting that isoegomaketone could be transformed to PK (Table 6). However, the enzymes involved in the transformation from PL to PK need to be elucidated.

For the EK biosynthesis, geranial (trans-citral) can generate neral (cis-citral) through structural changes, followed by the formation of naginataketone, which was speculated to be regulated by the genes N and elsholtziaketone was formed by the enzymes coding by genes P and Q, resulting in the production of the EK-type, with elsholtziaketone as the main component [80]. The SF-type is generated via the conversion of neral. These genes in the PK, EK, and SF biosynthetic pathways have not been elucidated (dotted arrows in Fig. 5).

### 5.2 Biosynthesis of PPs

In contrast to MTs, PPs were speculated to be synthesized in the absence of gene G and were controlled by the recessive gene g (Fig. 5) [81]. Biosynthetic studies on PPs are scarce, and the synthesis of these compounds was speculated to occur from L-phenylalanine via the shikimic acid pathway.

Elemicin was thought to be synthesized from methyl eugenol under the control of gene E to form the PP-e type. It was speculated that apiole, which has the same skeleton structure, was formed by methyl eugenol [78]. Only dillapiole and nothoapiole synthases have been preliminarily identified in the biosynthetic pathway of PPs. A P450 enzyme, CYP71D558 (GenBank Accession No. LC476554), which produced a dillapiole intermediate by introducing a hydroxy group to myristicin, was characterized in a study [30].

Baba et al. [31] characterized a hydroxylase, Pf-18428 (GenBank Accession No. LC596386), which was classified into the CYP71D subfamily and could synthesize an intermediate to produce nothoapiole using apiole and dillapiole as substrates.

### 5.3 Biosynthesis of other compounds

Along with the enzymes that participate in the biosynthesis of the compounds that determine *P. frutescens* chemotypes, some enzymes that catalyze the biosynthesis of some low-content compounds have also been identified. The myrcene synthase PTS-5526 (GenBank Accession No. AF271259) was cloned from a PK-type *P. frutescens* by Hosoi (Table 7) [80]. Besides being the first acyclic monoterpene synthase from *P. frutescens* whose functions have been characterized, it is also the first myrcene synthase cloned from Labiatae plants.

Linalool synthases were cloned from different chemotypes and have been functionally characterized in *E. coli* (Table 7) [[74], [75], [76]]. Linalool synthases share the same catalytic substrate, GPP, with other monoterpene synthases. As an important branch point in the first step of the biosynthesis of monoterpenes, it may act as a regulatory factor that is crucial for the formation of different chemotypes in *P. frutescens*.

The biosynthesis pathways of PA and C have been elucidated. However, some key enzyme-encoding genes involved in the biosynthesis of PT, PL, PK, EK, SF, PP-e, PP-as, and PP-a have not been fully characterized, and further researches are needed.

## 6 Discussion

It is common that a plant produces different types of metabolites. However, the occurrence of several chemotypes with only one or two dominant component(s) in one species is unusual. Within the *P. frutescens* population, strong genetic (dominant/recessive) relationships were found among the different chemotypes. The following speculations provide clues for studies on the molecular mechanism underlying these genetic relationships, which need to be elucidated.

What causes the diversity of *P. frutescens* chemotypes? The polyploidization [66] that occurred during the evolution of *P. frutescens* may strongly contribute to the diversity of its secondary metabolites and further diversity of its chemotypes. Many homologous genes, including those involved in the biosynthetic pathways of secondary metabolites, were generated in *P. frutescens*, a young allotetraploid species. The diversity of terpene synthases was resulted from the expansion of the TPS gene family in allotetraploid species [66].

The ecological adaptation of *P. frutescens* to the environment may lead to its diverse metabolites and chemotypes too. The *P. frutescens* plants are widely distributed in the world, while some chemotypes was only found in specific areas. For example, the collection of a large number of germplasm resources in the mountainous areas of northern Thailand found that most *P. frutescens* individuals were PT-type, but this chemotype was not found in Japan [64].

What is the mechanism underlying the formation of different chemotypes? In other words, why do similar amounts of different metabolites have difficulty coexisting in the same plant? Since the mechanisms contributing to chemotype formation among chemotypes whose dominant compounds are in the same biosynthesis pathway and those with dominant compounds from different pathways may differ, they were discussed separately here.

Take PL and PK which are monoterpenes in the same biosynthetic pathway as example (shown in Fig. 5). PL was believed to be the intermediate of PK. However, only a small amount of PL could be detected in PK-type *P. frutescens*. Once the intermediate is formed, it could be catalyzed immediately when the catalyzing enzymes are sufficient, until the generation of the final product. Thus, intermediate compounds are difficult to detect when they are present at low concentrations. This can explain the small amount of PL as an intermediate of PK in the PK-type *P. frutescens*.

On the other hand, the mutations of the enzyme-encoding genes that result in loss of function or loss of expression could lead to the accumulation of its substrate which is intermediate compounds of the original chemotype (such as PK-type). That will form a new chemotype with the intermediate (such as PL-type). The pseudogenization or loss of downstream genes in the biosynthetic pathway was attributed to the ability of Solanaceae plants to synthesize tropine [82]. The formation of chemotypes is also influenced by regulatory factors such as promoters. Differences in artemisinin yield among different *Artemisia* varieties may occur due to variations in promoter of a key gene [83].

The chemotypes may have main components synthesized through different branched pathways, such as PA-type and C-type (Fig. 5). They share the same catalyst precursor, GPP. Thus, the direction of downstream biosynthesis could be directly determined by the presence or absence of TPS genes. For example, the formation of six chemotypes in tea trees (*Melaleuca alternifolia*) can be explained by the “supergene structure”, which is a genetic model in which several key genes in a presence/absence variation in the biosynthetic pathway [84].

However, when multiple enzymes are present at the same time, the formation of chemotype might be associated with the affinity of different enzymes to the same substrate GPP, especially when the substrate is limited.

Moreover, the interactions between enzymes in metabolic pathways lead to the formation of enzyme complexes, which affect enzyme activities and the direction of metabolic pathways. Finally, other factors such as the expression levels and stability of different TPSs might also influence the direction of metabolic flux, thus affecting the formation of chemotype as well as the dominant or recessive inheritance of different chemotypes.

The asymmetric subgenomic evolution in *P. frutescens* was conspicuous, with intrachromosomal rearrangements of sub genome PFB being greater in number than those of sub genome PFA. Compared to PFB, PFA had a higher gene retention rate and expression but a lower pseudogenization rate [66], which indicated a higher gene content and greater contribution of the dominant PFA to global transcriptome profiling than the fractionated PFB. The distribution of genes and regulatory genes involved in the biosynthesis of specific chemotypes in the PFA or PFB subgenomes may determine whether it is a recessive chemotype.

Factors that mentioned above may be helpful in revealing the mechanism of chemotypes variation of *P. frutescens*.

## 7 Reflections on the development of *P. frutescens*

Several *P. frutescens* varieties with similar phenotypes are cultivated extensively in China, which makes accurate identification of chemotype difficult and threatens the drug safety. The chemotype of essential oil can serve as a key point for the intraspecific classification of *P. frutescens*, particularly for medicinal use.

Therefore, *P. frutescens* varieties which have high quality and are safe for medicinal purposes should be cultivated. The identification of chemotypes and evaluation of their inheritance, clarification of the mechanisms underlying the biosynthesis and regulation of the key compounds of essential oils, and improvement of the accumulation of these bioactive compounds via genetic engineering or gene editing technologies [85,86] may contribute to the germplasm innovation for such varieties and the improvement of the quality of *P. frutescens* related medicine.

The assembly of a high-quality, chromosome-scale-based genome of tetraploid *P. frutescens* has recently been performed via advanced long-read sequencing and chromosome conformation capture technologies [66], marking the entry of the medicinal *P. frutescens* plant into the postgenomic era.

Over the past decade, population genetics has been applied along with high-throughput sequencing in various studies for the genetic analysis of different important traits in different types of plants, which has led to significant progress. These studies may provide methodological guidelines for studying chemotype formation and its genetic regulatory mechanisms in *P. frutescens*. For example, loci related to 11 important agronomic traits of cotton were identified via a GWAS [87]. New technology could also be utilized to identify loci of metabolite related traits. High marker density GWAS provides new insights into the genomic architecture of terpene oil yield traits in *Eucalyptus* [88]. In tomato, a total of 610 accessions were collected, and multiple genetic loci associated with fruit traits were identified via mGWAS [89]. The investigations of the genetic resources underlying metabolites via mGWASs and mQTLs have also facilitated the large-scale identification of genes and the elucidation of pathways in tomatoes [90] and wheat [91]. These method have also been used to identified genes that related to the regulation of the amino acid content in tea trees [92] and malic acid metabolism related genes in grapes [93].

These methods are useful for further identifying the key genes associated with the formation of different chemotypes of *P. frutescens*; thus, further studies are necessary to understand its genetic and molecular mechanisms.

Secondary metabolites as the core components of medicinal plants play various roles. For innovations in the germplasm of medicinal plants, the preservation of their chemotype characteristics should receive special attention. Therefore, for domestication and germplasm innovation in *P. frutescens*, metabolomics should be applied along with molecular marker-assisted breeding. Through metabolome and transcriptome analysis in combination with whole-genome resequencing, researchers may be able to solve the mystery of chemotype inheritance in *P. frutescens* and promote molecular breeding of the medicinal *P. frutescens*.

## CRediT authorship contribution statement

**Wei Wei:** Writing – original draft. **Zhaoyuan Li:** Investigation, Data curation. **Bin Wang:** Resources, Investigation. **Yang Liu:** Methodology. **Yuxuan Sun:** Visualization, Investigation. **Dong Wen:** Investigation. **Mei Rong:** Software. **Pengcheng Huang:** Visualization, Investigation. **Yuwei Guo:** Data curation. **Qiuling Wang:** Supervision, Project administration, Funding acquisition. **Zhihui Gao:** Writing – review & editing. **Jianhe Wei:** Resources, Funding acquisition, Conceptualization.

## Declaration of competing interest

The authors declare that they have no known competing financial interests or personal relationships that could have appeared to influence the work reported in this paper.
